# An added dimension to tumour TRAIL sensitivity

**DOI:** 10.18632/oncoscience.267

**Published:** 2015-11-18

**Authors:** Sandra Healy, Lynda O'Leary, Eva Szegezdi

**Affiliations:** Apoptosis Research Centre, National University of Ireland, Galway, Ireland

**Keywords:** TRAIL, cancer, microenvironment, decoy receptors, 3D model

Tumour necrosis factor-related apoptosis-inducing ligand (TRAIL) is a member of the TNF cytokine family and a selective inducer of apoptosis in a range of tumour cells, but not in healthy normal, untransformed cells. It is expressed by natural killer cells and natural killer-T cells when they encounter malignantly transformed cells and it is a key effector molecule in tumour immune surveillance. TRAIL has 5 receptors, which is the highest receptor promiscuity in the TNF ligand family. It binds to death receptor 4 (DR4) or DR5 on the surface of target cells [1] and initiates a conformational change which promotes association of the receptors with FADD facilitating pro-caspase-8 and/or pro-caspase-10 recruitment which then activates effector caspases to execute cell death [2]. Signalling through DR4 and DR5 can also activate pro-inflammatory intracellular molecules such as MAPK, PKB and NF-κB and overexpression of DR4 or DR5 has been shown to stimulate the release of inflammatory cytokines [3]. However, TRAIL also has three regulatory receptors. Two of these, decoy receptor 1(DcR1) and DcR2 are membrane bound and the third regulatory receptor, osteoprotegerin is a secreted protein. DcRs regulate TRAIL-induced apoptosis by either sequestering TRAIL from the death receptors or by forming inactive, heteromeric DcR1/2–DR4/5 complexes [1]. Indeed, DcRs have been shown to be highly expressed in a number of tumour tissues such as acute myeloid leukaemia, prostate cancer and breast cancer and their expression is linked with poor prognosis [4]. However DcR expression in tumour cells does not correlate with TRAIL sensitivity and non-transformed cells do not require DcRs to be protected from TRAIL-induced apoptosis, suggesting that the *in vivo* role of the DcRs may be more complex than originally thought [5]

The tumor-specific cytotoxicity of TRAIL has been exploited as a therapeutic strategy by utilizing recombinant versions of TRAIL and agonistic antibodies against DR4 and DR5 [6]. While recombinant soluble human TRAIL was highly potent against a broad range of tumours in vitro and in pre-clinical studies, in clinical trials TRAIL has failed to exhibit the same potency [6]. One of the major shortcomings of the preclinical models was the lack of assessment of the contribution of the tumour microenvironment (TME). The TME consists of various cell types, soluble factors and signals from the extracellular matrix, and is in a reciprocal interaction with the tumour cells. It is thus important to understand the interplay of different cell types in the tumour microenvironment and the effect of the factors they express and secrete on tumour growth, development and resistance to therapy.

**Figure 1:**
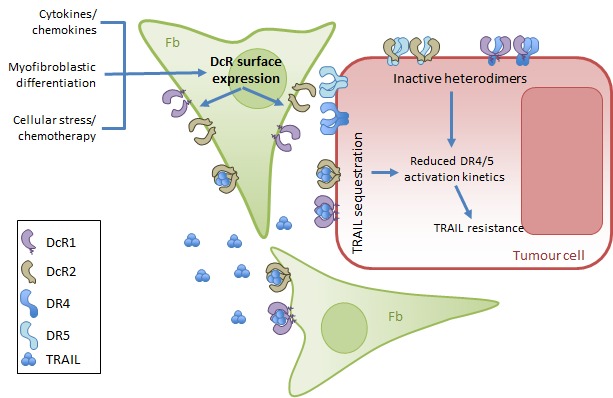
Cell autonomous and supracellular regulation of TRAIL-sensitivity by decoy receptor 1 (DcR1) and -2 Decoy receptors expressed by the tumour cells can drive cell autonomous (cis-) regulation of TRAIL-sensitivity. The expression of both DcR1 and -2 can be induced in the stroma in response to cellular stress (induced e.g. by chemotherapy), cytokines or myofibroblastic differentiation occurring during tumour pathogenesis. Stromal-expressed DcRs also sequester TRAIL; and similar to the cis-regulation, they reduce the kinetics of DR4/5-activation, resulting in TRAIL resistance. Fb: stromal fibroblast, DcR: decoy receptor, DR: death receptor

The study by O'Leary and colleagues explored the hypothesis that DcRs exerted a ‘supracellular level control’ of TRAIL-sensitivity rather than simply regulating TRAIL resistance at a cell-autonomous level [7]. They firstly examined the expression of the decoy receptors in tumour cells, tumour stroma and in non-malignant, tumour adjacent tissues. Interestingly they found that DcR1 and DcR2 are commonly expressed in tissues but tissue stroma only express DcR1. To determine whether DcR-expressing stromal cells influence the TRAIL sensitivity of tumour cells sharing the same microenvironment they generated and characterized DcR-insensitive TRAIL mutants and used a combination of mathematical modelling, cell based assays and a stroma/tumour co-culture system allowing them to model the effect of DcRs expressed by adjacent stromal cells on DR4/5 activation in tumour cells. They found that stromal DcRs profoundly reduced TRAIL-induced DR4/DR5 activation and protected tumour cells against TRAIL. The authors confirmed this finding in a 3D mixed-cell type (stroma-tumour) spheroid tumour model. Interestingly they also observed that TNF increased surface expression of the DcRs in fibroblasts raising the possibility that an inflammatory environment may induce DcR expression and promote TRAIL resistance. This study clearly demonstrates that stromal DcRs in the tumour microenvironment can exert trans-cellular regulation affecting tumour cells and it highlights the importance of developing therapeutic TRAIL variants that can selectively activate the two death inducing TRAIL receptors but are not mopped up by the decoy receptors present on stromal tissues. Future studies will hopefully establish a feed forward signalling loop that drives overall tissue sensitivity in order to achieve tumour eradication.
